# Head and neck cancer exosomes drive microRNA-mediated reprogramming of local neurons

**DOI:** 10.20517/evcna.2020.04

**Published:** 2020-12-30

**Authors:** Patrick J. Hunt, Moran Amit

**Affiliations:** 1 Medical Scientist Training Program, Baylor College of Medicine, Houston, TX 77030, USA; 2 Department of Neurosurgery, Division of Surgery, The University of Texas MD Anderson Cancer Center, Houston, TX 77030, USA; 3 Department of Head and Neck Surgery, Division of Surgery, The University of Texas MD Anderson Cancer Center, Houston, TX 77030, USA

**Keywords:** MicroRNA, microenvironment, adrenergic neurons, solid tumors, neurotrophic growth, neuron-tumor crosstalk

## Abstract

Solid tumors are complex collections of cells surrounded by benign tissues that influence and are influenced by the tumor. These surrounding cells include vasculature, immune cells, neurons, and other cell types, and are collectively known as the tumor microenvironment. Tumors manipulate their microenvironment for the benefit of the tumor. Autonomic neurons innervate and drive malignant growth in a variety of solid tumors. However, the mechanisms underlying neuron-tumor relationships are not well understood. Recently, Amit *et al.* described that trophic relationships between oral cavity squamous cell carcinomas (OCSCCs) and nearby autonomic neurons arise through direct signaling between tumors and local neurons. An inducible tumor model in which 4NQO was introduced into the drinking water of *Trp53* knockout mice was used to model OCSCC-microenvironment interactions. Using this model, this group discovered that loss of p53 expression in OCSCC tumors resulted in increased nerve density within these tumors. This neuritogenesis was controlled by tumor-derived microRNA-laden extracellular vesicles (EVs). Specifically, EV-delivered miR-34a inhibited neuritogenesis, whereas EV-delivered miR-21 and miR-324 increased neuritogenesis. The neurons innervating p53-deficient OCSCC tumors were predominantly adrenergic and arose through the transdifferentiation of trigeminal sensory nerve fibers to adrenergic nerve fibers. This transdifferentiation corresponded with increased expression of neuron-reprogramming transcription factors, including POU5F1, KLF4, and ASCL1, which were overexpressed in the p53-deficient samples, and are proposed targets of miR-34a-mediated regulation. Human OCSCC samples enriched in adrenergic neuron markers are associated strongly with poor outcomes, thus demonstrating the relevance of these findings to cancer patients.

Cancers are predominantly characterized by their loss of proliferative control. Genetic changes drive this increased proliferation, resulting in the formation, growth and spread of tumor cells throughout the body. Mutations that commonly drive tumor growth, however, also drive molecular changes within the tumor and the surrounding tissues. Encircling the solid tumors are collections of healthy cells that typically act to support cell types and tissue functions in the local region. These cells include structural fibroblasts, immune cells, neurons, blood vessels, and other cell types that in combination are known as the tumor microenvironment. In the context of cancer, these supporting cells can be manipulated to support the growth and spread of the tumor. These manipulative relationships become essential for the survival of the cancer and are found in prostate^[1,2]^, gastric^[3–5]^, pancreatic^[6]^, skin^[7,8]^, glioma^[9–11]^, and a variety of other tumor types^[12–15]^. Subsequently, efforts to interrupt the relationships between solid tumors and immune cells^[16]^, as well as between tumors and blood vessels^[17]^ have shown efficacy in stymying tumor growth, demonstrating the widespread therapeutic potential borne from understanding these tumor-microenvironment relationships. To date, the relationships between tumors and other members of the tumor microenvironment are not well understood. Additionally, the mechanisms by which these relationships are formed and sustained are not well documented. Recent work from Amit *et al.*^[18]^ has uncovered that tumors use extracellular vesicular signaling to drive nearby neuron survival, growth, spread, and subtype switching, which, in turn, drives tumor growth.

This recent work focused on oral cavity squamous cell carcinoma (OCSCC), an aggressive tumor arising from mouth epithelial cells. To study these tumors in greater depth, the group leveraged laboratory mouse models of OCSCC. These include mice in which the tumor suppressor p53 was conditionally knocked out of epithelial cells (*Krt5^cre^; Tp53^flox/flox^*). The control group for these mice lacked the *Krt5^cre^* allele, leaving *Tp53* intact in all cells. In these models, tumor initiation was induced via introduction of the carcinogen, 4-nitroquinoline 1-oxide (4NQO) into the drinking water. Additionally, the group used patient-derived xenograft models in which *p53^WT^* or *p53^null^* OCSCC cells were injected into the tongues of mice and allowed to grow. Finally, in cell culture dishes, dorsal root ganglia (DRG) were co-cultured with oral keratinocytes and *p53^WT^* or *p53^null^* OCSCC cells to model neural interactions with OCSCC tumors *in vitro*.

Initially, the group found that in tumor samples derived from both their conditional *p53* knockout mice, as well as their patient-derived xenograft models, loss of *p53* coincided with increased adrenergic nerve density within the tumor. These findings were recapitulated in human OSCSS samples, indicating that tumor cells lacking *p53* were driving increased adrenergic neuritogenesis. When testing this same hypothesis using *in vitro* OCSCC-DRG co-cultures, Amit *et al.*^[18]^ concordantly found increased DRG innervation of *p53^null^* OCSCC cells. When *p53^WT^* OCSCC-DRG co-cultures were incubated with conditioned medium containing extracellular vesicles (EVs) from *p53^null^* OCSCC cells, the same neuritogenic effect was observed. Additionally, when the *p53^null^* OCSCC cells co-cultured with DRGs were inhibited from releasing EVs, the effect was lost, thus demonstrating that the *p53^null^*-derived EVs and their contents were the driving force behind the described neuritogenic effect.

Extracellular vesicles serve as important vehicles of small regulatory RNA species, which are known to be essential for proper neuronal development and function. By comparing the small RNAs found within EVs derived from *p53^WT^* and *p53^null^* cell lines, Amit *et al.*^[18]^ found that 17 microRNAs (miRNAs) were down-regulated in *p53^null^* cells. After narrowing this collection of miRNAs to the most down-regulated species, they found that several of these miRNAs, including miR-34a, were also down-regulated in the *p53^null^* tumors found within *in vivo* mouse models. Thus, these data suggest that miR-34a, and other similarly acting miRNAs, prevent neuritogenesis and that, when lost in *p53^null^* EVs, the surrounding neurons exhibit increased neuritogenesis. In concert with this hypothesis, the group next inhibited miR-34a by transfecting cultured neurons with an antagomiR that specifically inhibits miR-34a. This experiment resulted in increased neuritogenesis in the antagomiR-34a transfected neurons when compared to neurons transfected with scrambled antagomiRs, thus confirming the hypothesis that miR-34a from *p53^WT^*-derived EVs acts to prevent neuritogenesis.

To complement these findings, they next identified candidate miRNAs that drive neuritogenesis. Towards this effort, they uncovered that antagomiR-mediated inhibition of miR-21 and miR-324 resulted in decreased neuritogenesis in transfected neurons when compared to those transfected with scramble antagomiRs. Conversely, transfection of neurons directly with a combination of miR-21 and miR-324 resulted in a robust increase in neuritogenesis when compared to neurons transfected with scramble miRNA molecules. Moreover, adding miR-34a to this combination of miR-21 and miR-324 decreased neuritogenesis, demonstrating that cancer-driven neuritogenic processes lie in a delicate balance, dictated, at least in part, by these miRNAs.

The researchers noticed that these newly formed neurites stained positively for the adrenergic marker tyrosine hydroxylase (TH), demonstrating thereby that the responding neurons were adrenergic in nature. However, upon exposure to EVs derived from *p53^null^* OCSCC tumors, they found that the number of adrenergic neurons increased dramatically in both the *in vitro* and *in vivo* models, suggesting that *p53^null^*-derived EVs were promoting an adrenergic state. Conversely, exposure to *p53^WT^*-derived EVs decreased the number of intratumoral TH-positive adrenergic neurites, suggesting that these wildtype EVs inhibit cancer-associated adrenergic neuritogenesis. At this point, the researchers were uncertain about the origin of these adrenergic neurites. Amit *et al*.^[18]^ wanted to know whether these adrenergic neurites arose from previously existing adrenergic neurons or whether these newly-formed adrenergic neurites arose from another neuron type. They subsequently found that transfection of trigeminal sensory neuronal cultures with miR-21 and miR-324 resulted in increased adrenergic staining, suggestive of neurotype switching from a sensory to a sympathetic nature. However, when miR-34a was added to the combination of miR-21 and miR-324, the effect was lost, demonstrating that along with inhibiting neuritogenesis, miR-34a activity also inhibits neo-adrenergic neurotype switching. These findings were subsequently bolstered by transcriptomic analysis, which demonstrated that neurons incubated with EVs derived from *p53^null^* OCSCC tumors expressed increased levels of catecholamine biosynthesis-related genes and decreased levels of sensory neuron signaling genes. Specifically, the transcription factors POU5F1 and KLF4 were found to be upregulated in trigeminal neurons following incubation with EVs derived from *p53^null^* OCSCC tumors. These transcription factors are sufficient to drive neuronal differentiation of adult neural stem cells. Additionally, these two factors are directly regulated by miR-34a activity. NEUROG2 and ASCL1 are two additional factors that were upregulated in trigeminal neurons following incubation with *p53^null^* OCSCC EVs. These factors are also candidate targets of miR-34a regulation, and their activity drives neuronal differentiation, specifically to an adrenergic fate. These expression changes illustrate a neurotype switching event in sensory neurons adjacent to *p53^null^* OCSCC tumors. Moreover, the combination of the findings described thus far suggest that depletion of miR-34a in EVs released by *p53^null^* OCSCC tumors is the mechanism by which these tumors drive increased neo-adrenergic innervation of the tumor body.

Knowing that the tumor-innervating neurons that drive neurotropic tumor growth are adrenergic in nature opens up avenues for the use of readily available therapies to treat patients with OCSCCs. Beta adrenergic receptor blocking medications, for example, are already approved for the treatment of hypertension, heart arrhythmias, angina, migraines and other illnesses, and are widely available^[19]^. Additional data from published clinical trials support the use of anti-adrenergic approaches to treating several types of cancers, including breast cancer and hepatocellular carcinoma, among others^[20–25]^. Additionally, several ongoing clinical trials are testing anti-adrenergic medications as an adjuvant or stand-alone therapy in prostate and pancreatic cancers (NCT03152786, NCT02944201, NCT03838029, NCT04245644). Within this study, Amit *et al*.^[18]^ found that Carvedilol, which non-selectively blocks α1, β1, and β2 adrenergic receptors, dramatically decreased tumor growth and proliferation rates when compared to tumors within vehicle-treated mice harboring patient-derived xenografted *p53^null^* OCSCC tumors. Supporting the use of anti-adrenergic therapies in humans, this group found that the level of TH staining in human OCSCC samples was an independent predictor of increased tumor recurrence and decreased patient survival. Thus, this work argues for an increased focus on anti-adrenergic approaches for treating OCSCC as well as other cancers.

Similar to the findings by Amit *et al*^[18]^, Magnon *et al*.^[1]^ found an increased density of TH-positive adrenergic neurons within the tumor microenvironments of patients with high-risk prostate adenocarcinomas^[1]^. This group showed that the number of adrenergic neurons within the tumor microenvironment was an independent predictor of tumor recurrence. They also found a similar relationship between tumor aggression and the amount of parasympathetic vesicular acetylcholine transporter (VAChT)-positive staining within the tumor body. However, they were not able to determine whether this increase in autonomic innervation was due to neurogenesis, neuritogenesis, neuron-type switching, or some other mechanism. Additionally, they were not able to determine if the tumor was driving this increase in autonomic neurite number, and by what means these aggressive tumors were communicating with local neurons. In contrast, the findings presented by Amit *et al.*^[18]^ shed light on possible mechanisms of communication between prostate tumors and local innervating autonomic neurons. These experiments add to the already rich literature describing the role that EVs play in regulating the tumor microenvironment and cancer metastasis. Future work in prostate adenocarcinoma should examine the EVs released by these tumors, and the miRNA contents that may be manipulating local neurons. A recent report systematically demonstrated the importance of EV contents in the development and growth of a variety of cancers^[26]^. Though small RNA messengers and regulators were found to be important in mediating tumor growth in this study as well, this group provided ample evidence supporting damage-associated molecular patterns (DAMPs) and other cancer-associated proteins as essential mediators of neuron-tumor trophic interactions. These findings point to the possibility that protein messengers are important in establishing and maintaining the trophic relationships between prostate adenocarcinomas and local autonomic fibers, as well as *p53^null^* OCSCC tumors and local sensory/ adrenergic neurons.

Though the mechanisms governing neuron-tumor relationships described by Amit *et al.*^[18]^ were worked out between OCSCC tumors and sensory/adrenergic neurons of the oral cavity, many of these principles might be generalizable to other tumor types and neuronal subtypes. Already, increased neuritogenesis and sensory-autonomic neurotype switching has been reported in pancreatic nerves in the context of pancreatic cancer^[27]^. However, the findings presented here also differ from those published in previous work. In contrast to the findings of Magnon *et al.*^[1]^, Amit *et al*.^[18]^ found no increase in parasympathetic VAChT-positive neurites in p53^null^ OCSCC tumors. Thus, it is possible that for each tumor type and each region of local autonomic fibers, a balance of sympathetic and parasympathetic neurons guides tumor growth and survival. Future work will need to examine these balancing forces within each tumor type and the corresponding microenvironment.

This work describes novel mechanisms of EV-mediated regulation of tumor activity, which highlights new hypotheses regarding the biology underlying these mechanisms. Differential delivery of miR-34a *vs*. miR-21 and miR-324 to local neurons could be the result of differential loading of miRNAs into EVs. Alternatively, the differing contents of these cancer-derived EVs could be dictated strictly by expression changes within p53^null^ and p53^WT^ cancer cells. Specific analysis of transcriptional changes in p53^null^ and p53^WT^ cancer cells, combined with detailed profiling of EV contents will allow for a deeper understanding of the mechanisms governing this described specificity of EV contents. In tandem with this, specific subpopulations of p53^null^ and p53^WT^ tumor cells likely contribute more to the specific release of different EV-delivered miRNAs than other subpopulations of p53^null^ and p53^WT^ tumor cells. Cellular heterogeneity within tumors is a well-described aspect of tumor biology that affects tumor activity, and the different mutations and transcriptional profiles found within different subpopulations may be an important driver of differential miRNA expression and release through EVs. Modern sequencing techniques, including single-cell RNA sequencing of tumor samples will be valuable in unraveling the link between specific transcriptional profiles and EV-mediated miRNA delivery. Additionally, this work argues for the increased use of EV sampling in the diagnosis, surveillance and treatment of various cancer types. Sampling EV contents from blood, plasma, and other bodily fluids is a non-invasive and cost-effective method of providing researchers and care teams with myriad signals and transporters shared between the tumor and the microenvironment^[28–31]^. Moreover, while these contents serve as potent biomarkers of cancer presence, diagnosis, and progression, they also provide clues as to which treatment approaches may be most effective in treating specific patients. Stemming from this work, analysis of EV contents will also identify novel proteins and RNA messengers, thereby identifying alternative signaling pathways, and furthering our understanding of how neuron-tumor trophic relationships are established and sustained.

In this work, Amit *et al.*^[18]^ uncovered a novel and potent method by which OCSCC tumors manipulate nearby sensory neurons to drive a neo-adrenergic neurotype switching event, thereby driving tumor growth and spread. These findings constitute a paradigm shift in our understanding of how tumors and neurons within the microenvironment interact with each other. Moreover, this work illustrates the importance of EV contents, and more specifically, miRNA signaling in mediating the trophic relationships between tumors and neurons that drive tumor growth. Future work will focus on further deciphering EV-mediated signaling, with a focus on developing novel approaches of using EV analysis to better understand tumor biology, and to better treat human cancer patients.
